# Increased Light Extraction of Thin-Film Flip-Chip UVB LEDs by Surface Texturing

**DOI:** 10.1021/acsphotonics.2c01352

**Published:** 2023-01-24

**Authors:** Michael A. Bergmann, Johannes Enslin, Martin Guttmann, Luca Sulmoni, Neysha Lobo Ploch, Filip Hjort, Tim Kolbe, Tim Wernicke, Michael Kneissl, Åsa Haglund

**Affiliations:** † Chalmers University of Technology, Department of Microtechnology and Nanoscience, 41296Gothenburg, Sweden; ‡ Technische Universität Berlin, Institute of Solid State Physics, 10623Berlin, Germany; § Ferdinand-Braun-Institut gGmbH, Leibniz-Institut für Höchstfrequenztechnik, 12489Berlin, Germany

**Keywords:** light-emitting diode, AlGaN, ultraviolet, electrochemical etching, surface texturing, light extraction

## Abstract

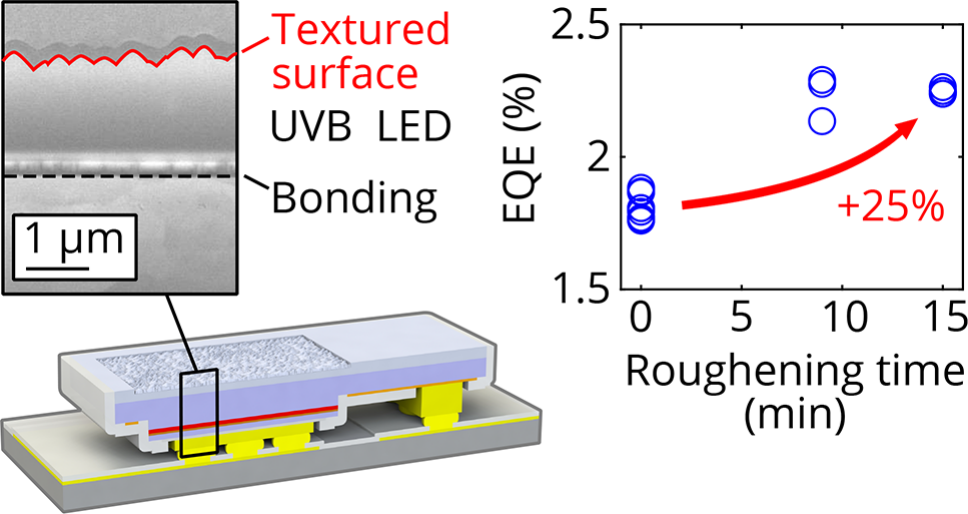

Ultraviolet light-emitting diodes (LEDs) suffer from a low wall-plug efficiency, which is to a large extent limited by the poor light extraction efficiency (LEE). A thin-film flip-chip (TFFC) design with a roughened N-polar AlGaN surface can substantially improve this. We here demonstrate an enabling technology to realize TFFC LEDs emitting in the UVB range (280–320 nm), which includes standard LED processing in combination with electrochemical etching to remove the substrate. The integration of the electrochemical etching is achieved by epitaxial sacrificial and etch block layers in combination with encapsulation of the LED. The LEE was enhanced by around 25% when the N-polar AlGaN side of the TFFC LEDs was chemically roughened, reaching an external quantum efficiency of 2.25%. By further optimizing the surface structure, our ray-tracing simulations predict a higher LEE from the TFFC LEDs than flip-chip LEDs and a resulting higher wall-plug efficiency.

Light-emitting diodes (LEDs) emitting in the ultraviolet (UV) range have applications such as water disinfection, sterilization, resist curing, and medical treatments.^1^ In comparison to the prevalent mercury-based UV lamps, AlGaN-based UV LEDs are environmentally friendly, can be tailored for a specific application, and their form factor allows for a convenient integration.^2^ However, the low wall-plug efficiency (WPE) below 10% for UV LEDs with an emission wavelength below 350 nm still limits their use.

In the UVB range (280–320 nm), the external quantum efficiency (EQE) is affected by the low internal quantum efficiency due to the high threading dislocation density from the growth on AlN/sapphire templates and the involved relaxation process due to low Al-contents.^3^ Recent improvements in injection efficiency and light extraction efficiency have recently resulted in an EQE of almost 10%.^4^ The main limiting factor for the EQE is the light extraction efficiency (LEE), which is therefore the most important to improve.

UV LEDs commonly employ a flip-chip (FC) LED design in which the light extraction through the sapphire backside is impeded by total internal reflection at the AlN/sapphire interface.^5^ Removing the substrate of flip-chip bonded LEDs reduces the number of interfaces and allows practicable texturing of the exposed AlGaN surface which increases the LEE.^5^ Such a thin-film flip-chip (TFFC) design, shown in Figure 1b, is commenly used for highly efficient GaN-based blue-emitting LEDs and can yield a very high LEE.^6,7^

**Figure 1:**
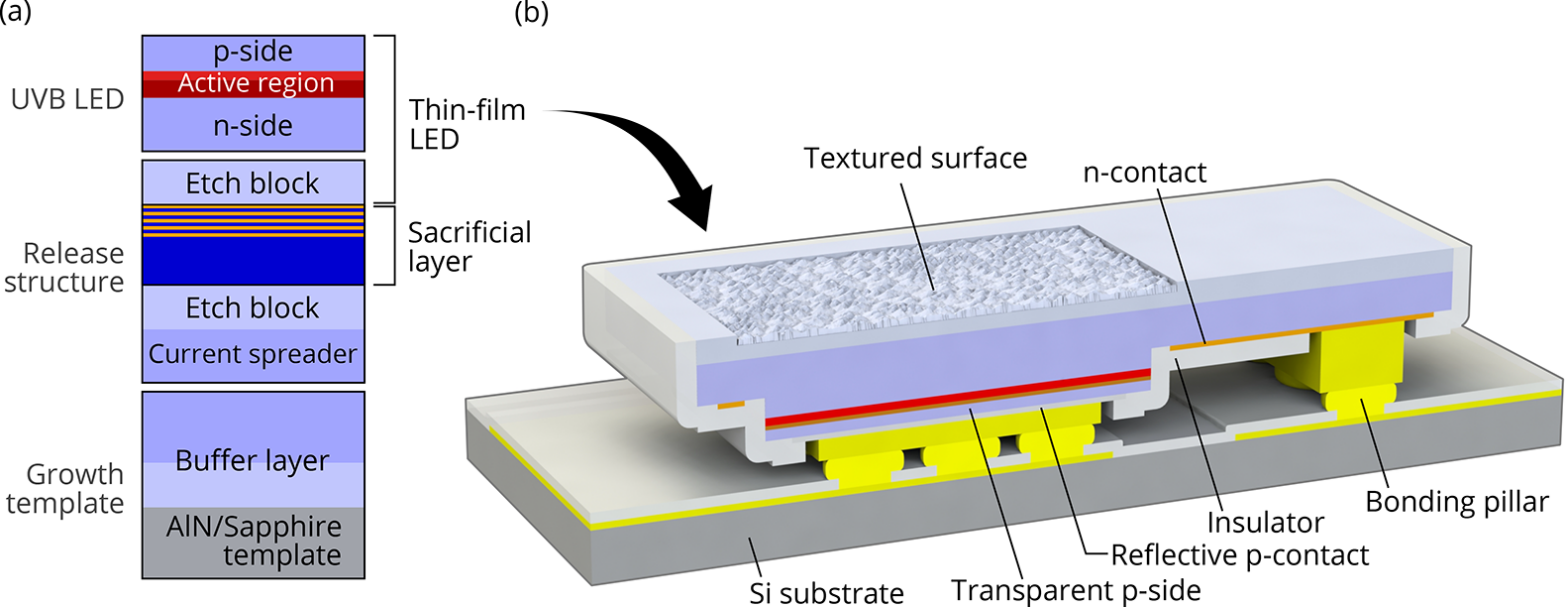
(a) Building blocks of the epitaxial structure and (b) schematic design of a TFFC LED with a roughened N-polar AlGaN surface.

TFFC UV LEDs have been demonstrated,^8−14^ and a performance improvement factor of 1.7^9^ and 1.31^13^ by using a TFFC design has been shown. However, the realization remains difficult because the thermal decomposition of AlGaN by the laser-lift off (LLO) for substrate removal results in Al residues as well as potential cracking of strained epitaxial layers.^11^ In recent work, this has been improved by growing on nanopatterned AlN and by polishing the surface after the LLO to remove metal residues and surface cracks. This is however challenging to do uniformly on strained wafers with bow.^15,16^ Alternatively, to circumvent LLO, UVB LEDs have been grown on SiC substrates to allow substrate removal by polishing and dry etching.^17^

Recently, we have shown an alternative method for the substrate removal, namely selective lateral electrochemical etching of AlGaN sacrificial layers^18^ and realized a proof-of-concept TFFC UVB LED.^19^ However, in these devices the n-contact was deposited after electrochemical etching to prevent the contact from being attacked during the electrochemical etching. This prevented annealing of the n-contact which resulted in poor IV-characteristics. In this paper, we demonstrate that it is possible to underetch fully processed LEDs including n-contacts, i.e., to combine standard LED processing with substrate removal based on electrochemical etching. This however requires the right epitaxial design of the sacrificial layer and etch block layers combined with proper device layout including protection layers. To further improve the devices, the exposed N-polar surface after substrate remove is chemically roughened.

The building blocks of the epitaxial structure are shown in Figure 1a, and the full structure is further described in Supplementary Note 1. The UVB LED with a p-side, transparent to the emitted light is grown on top of a sacrificial multilayer (a 118 nm thick Al_0.37_Ga_0.63_N layer and a five-period structure of alternating 5 nm thick Al_0.11_Ga_0.89_N and 5 nm thick Al_0.37_Ga_0.63_N), which is used to separate processed LED devices from the substrate later in the process. That multilayer is embedded between two etch block layers (uid and low-doped Al_0.50_Ga_0.50_N) to confine the electrochemical etching and prevent parasitic etching.^19^ The integration of a low-Al-content and high n-doping in the multilayer in combination with the unintentional doping of the top etch block layer and a Si concentration that is lower than that of the sacrificial layer, in this case 2 × 10^18^ cm^–3^ in the n-side of the LED, maximizes the etching selectivity. The sacrificial layer design with thin alternating low and high Al-contents allows for the use of lower Al-contents without degrading the crystal quality due to relaxation, which would happen in the case of a bulk layer. A low Al-content improves the etch selectivity between the sacrificial layer and the etch block layers, a selectivity that is further enhanced by the generated sheets of high carrier concentrations in the periodic structure created by the built-in polarization fields. Uniform electrochemical etching across the sample is achieved by including an n-doped current spreading layer below the bottom etch block layer.

The fabrication of the TFFC LEDs is described in detail in Supplementary Note 2. It starts by a standard LED process with mesa definition, metalization on the n- and p-side, and deposition of a passivation layer. The LED structure and the metal contacts are then fully enclosed with an additional resist layer to prevent parasitic etching during the subsequent removal of the sacrificial layer using electrochemical etching. In the next step, the LEDs are transferred using thermocompression bonding to Si carrier chips. The electrochemical etching and device transfer do not adversely affect the electrical properties of the LEDs, as shown in Supplementary Note 3.

TMAH-based wet etching is used to roughen the exposed N-polar AlGaN surface for 9 and 15 min to achieve different degrees of surface roughening and to investigate its impact on the light extraction. Figure 2a shows an as-transferred TFFC LED, whereas Figure 2b shows a device after 9 min roughening and Figure 2c shows a device after a 15 min roughening. Figure 2d shows an LED array with different degrees of roughening.

**Figure 2:**
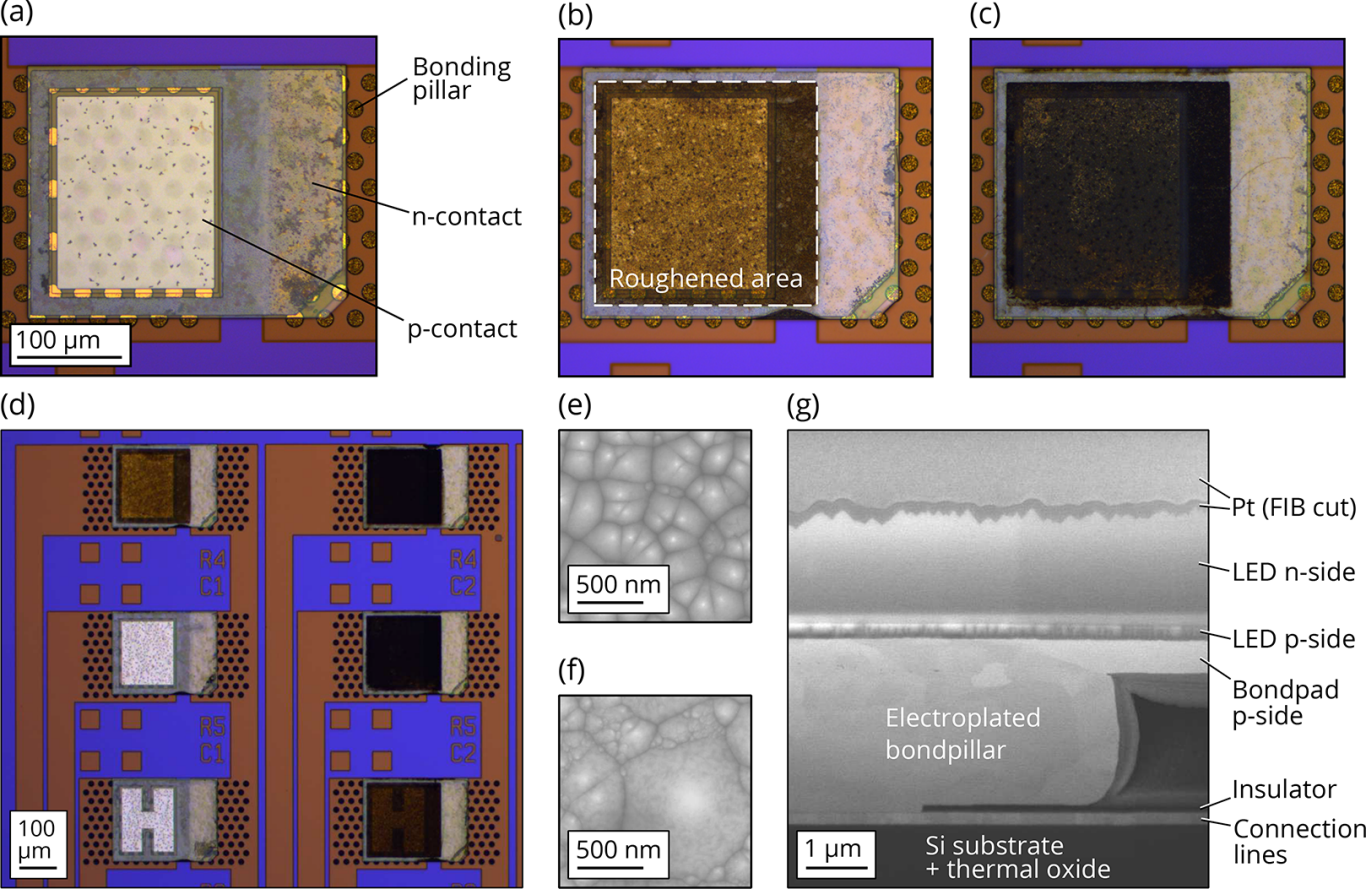
Microscope images of (a) an as-transferred LED, (b) an LED wet-etched for 9 min, and (c) an LED wet-etched for 15 min. (d) A TFFC LED array after roughening of selected devices for both 9 and 15 min. Top-view SEM images of the N-polar Al_0.5_Ga_0.5_N surface after (e) 9 min and (f) 15 min roughening. (g) SEM cross-sectional view of roughened TFFC LED.

Cross-sectional scanning electron microscopy (SEM) images of fabricated TFFC LEDs, see Figure 2g, show no parasitic electrochemical etching of the epitaxial layers, which indicates an appropriate choice of the etching conditions in combination with sacrificial, etch block and device layers. The cones of the roughened surface have an estimated cone angle of around 55^◦^ to 58^◦^, which is similar to values reported in previous works on roughened GaN^20^ and AlN.^21^ Top view SEM images of the roughened surface show a uniform cone distribution for 9 min roughening, see Figure 2e. For a longer etching time of 15 min, as shown in Figure 2f, larger cones evolve and reach lateral sizes of 1 μm, but the size distribution gets nonuniform.

The peak wavelength of the electroluminescence spectrum for the as-grown, as-transferred, and roughened LEDs is within 306 ± 2 nm, as seen in Figure 3a, which indicates no major strain change during substrate removal and roughening. A similar variation in the peak wavelength is obtained across the as-grown wafer suggesting local inhomogeneities to be the cause of the small variation in peak wavelength between the devices. In addition, the full width at half-maximum is 10 ± 1 nm for all devices and above 320 nm the spectra of the roughened devices show a low parasitic luminescence.

**Figure 3:**
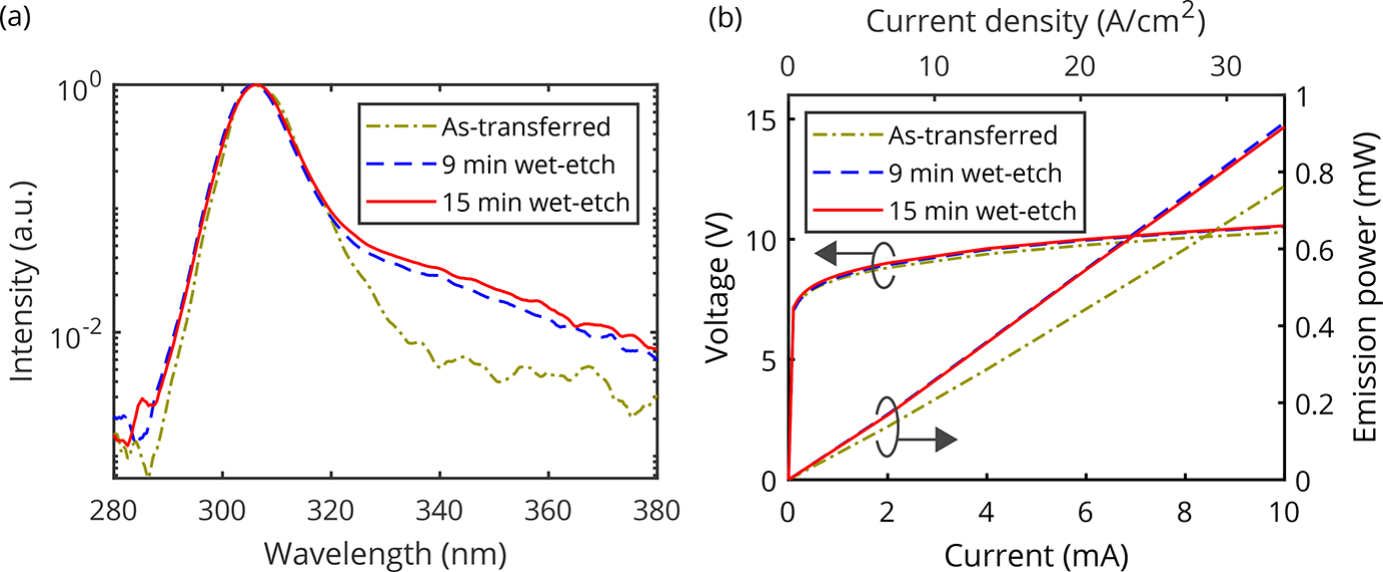
(a) Normalized electroluminescence spectra for as-grown, as-transferred, and roughened devices. (b) *L*–*I*–*V* characteristics for an as-transferred LED and two LEDs roughened for 9 and 15 min.

Figure 3b shows the *L*–*I*–*V* characteristics for an as-transferred device and two roughened devices after 9 min and 15 min roughening. The emission power of the device that has been roughened for 9 min has increased by around 24%. For 15 min roughening, there is no general improvement in emission power compared to 9 min roughening. This is attributed to a more inhomogeneous distribution in cone size which includes not only larger-sized cones but also smaller-sized ones compared to the 9 min roughened sample, as seen in the top view SEM images in Figure 2e,f. The *I*–*V* characteristics do not degrade by the roughening, as shown in Figure 3b. The maximum emission power of all fabricated devices is compared in Figure 4, which yields an average enhancement of 24% for 9 min and 25% for 15 min roughening. The surface roughening increases the maximum external quantum efficiency from 1.81% for as-transferred devices to 2.23% after a 9 min wet etching and 2.25% after a 15 min wet etching. Overall, a maximum wall-plug efficiency of 1.21% for 15 min of roughening is achieved.

**Figure 4:**
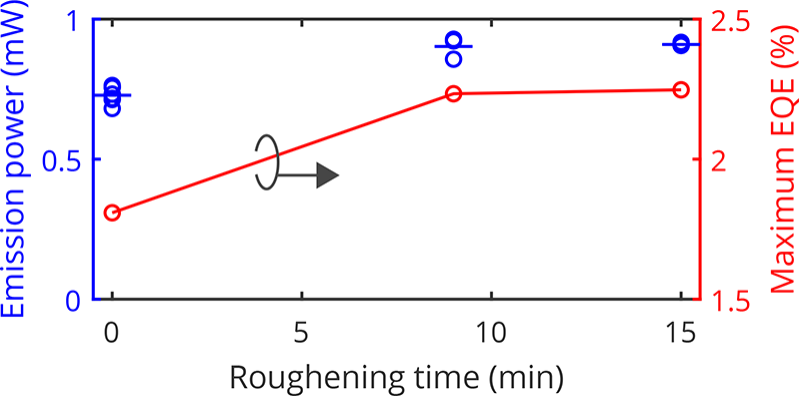
Emission power and the resulting maximum EQE as a function of the roughening time.

The surface morphology including cone angle and density was used to simulate the LEE for the fabricated devices and was compared to the measured enhancement. The calculation of the LEE is based on Monte Carlo ray-tracing simulations taking into account the refractive indices of the AlGaN layers,^22^ the sapphire substrate,^23^ and the SiO_2_ insulator,^24^ a dominating transverse-electric polarized emission pattern from the active region (with an assumed degree of polarization of 0.8, confirmed by measurements on similar UVB LED structures), the reflectivity of the contact metals, and the roughness of the interfaces.^25^ The absorption within the InAlGaN MQW was assumed to be 10^3^ cm^–1^ and for both the n- and p-AlGaN layers 10 cm^–1^ was used.^5,26^ The reflectivity of the Pd/Al/Ni/Au p-contact was assumed to be 60%^25^ and that of the V/Al/Ni/Au n-contact 20%.^27^ The roughness of the chip backside is based on a model which takes into account the distribution of the surface inclination angles that change the refraction, i.e., the ray path through the interface.^27^ As exemplarily shown in Figure 5a, based on SEM images (Figure 5a, 1.) a fictive height map was generated using randomly distributed cones with a slope angle of 58° and various cone densities (Figure 5a, 2.). As nanoscopic structures on the backside can be smaller than the wavelength of light, wave-optic effects are taken into account by using a Gaussian filter with a width of 450 nm on the height map leading to an effectively smoother interface (Figure 5a, 3.), i.e., a smaller average optical surface inclination angle that was calibrated by measuring the far field of collimated lasers before and after passing through a sapphire wafer with known roughness.^27^ The smoothed height map was then translated into an inclination angle (ϕ) histogram using the expression . The histogram is shown in (Figure 5a, 4.). From the histogram, the average optical surface inclination angle was calculated using
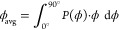
1where *P*(ϕ) is the probability density of the inclination angle ϕ. In this example, the average optical surface inclination angle is 19°, i.e., despite a facet angle of 58°, the nanosized cones exhibit a much smaller average optical inclination angle. However, a decreasing cone density would lead to larger average optical inclination angles and vice versa.

**Figure 5:**
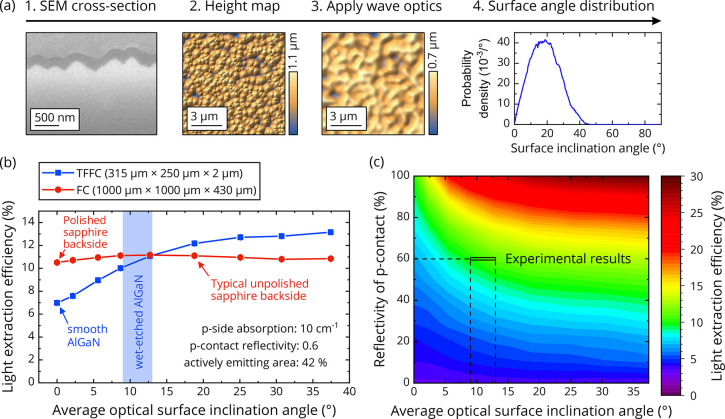
(a) Approach to model the roughened surface, (b) simulated light extraction efficiency of TFFC LED and FC LED with different degrees of roughness, and (c) simulated light extraction efficiency of TFFC LED with different p-contact reflectivities and degrees of roughness.

The simulated LEE of a FC and a TFFC LED is shown in Figure 5b as a function of the average surface inclination angle. The size of the simulated TFFC LED was 315 μm × 250 μm × 2 μm in comparison to a FC LED, which was assumed to be 1000 μm × 1000 μm × 430 μm in size, both with an emitting area of 42% of the lateral chip size. If the backside of the chip is smooth, i.e., an average surface inclination angle of 0°, the LEE of the FC-LED (10.5%) is higher than the LEE of the TFFC-LED (7%) due to the additional light extraction through the sapphire sidewalls in case of the FC design, while the TFFC design acts as a surface emitter. When the average surface inclination angle increases, the LEE increases and reaches a maximum of 11.2% at 13° for the FC LED and further increases to 13.2% at 37° for the TFFC LED. In the case of the FC design, the roughening of the sapphire backside has only a minor influence on the LEE due to the still flat AlN/sapphire interface. In the case of the TFFC design, the roughening of the AlGaN backside has a major influence on the LEE due to the increased probability of multiple reflections and light extraction. The current wet-etch process results in a cone density of 30 to 15 μm^–2^, which translates into an average surface inclination angle in the range of 9° to 13° (blue area in Figure 5b), and therefore, the LEE of the roughened TFFC LEDs is lower than what is expected from the conventional FC design.

Moreover, for such an average surface inclination angle, the simulations predict a 50% higher LEE compared to a device with an atomically smooth surface, i.e., a LEE of about 10–11% instead of 7%. Fabricated devices show a smaller improvement of about 25% but follow the trend of the simulations. This deviation is attributed to differences between the modeled and the real devices, e.g., an inhomogeneous distribution in cone size for the fabricated devices as well as a non atomically smooth surface of the unroughned devices due to the morphology of the as-grown material and electrochemically etched surface. However, with an increasing average surface inclination angle, i.e., decreased cone density and increased cone size, the TFFC LEDs’ LEE has the potential to exceed that of FC LEDs. Increasing the cone size requires more optimization of the wet etching conditions or an alternative texturing method such as a combination of dry and wet etching.

The light extraction for the TFFC LEDs further increases with a higher p-contact reflectivity, as shown in Figure 5c. Highly reflective p-contacts and a large surface roughness lead to an LEE above 25%. For such high LEE values, the actual LEE can be expected to be even higher as absorption and re-emission of photons in the active region, photon recycling, becomes significant and needs to be taken into account for an accurate modeling.

We have demonstrated thin-film flip-chip UVB LEDs using a lift-off technique based on lateral electrochemical etching to separate fully processed devices from the growth substrate. The sacrificial layer consisted of a multilayered structure, which enhanced the etch rate contrast to the surrounding layers, and combined with a suitable sample design with protective layers allowed for a reliable lift-off without parasitic etching. As a result, the electrical characteristic of the LED was not degraded by the electrochemical etching and bonding. The light extraction efficiency was increased when roughening the N-polar AlGaN surface of the TFFC LEDs, which increased the emission power by about 25% without degrading the *I*–*V* characteristics, resulting in an external quantum efficiency of 2.25%. Light-extraction simulations predict that an optimized roughening of the TFFC LEDs can further enhance the optical output power, but this would require an improved texturing method. Our TFFC LEDs demonstrate the potential of using electrochemical etching for substrate removal and a way forward to boost the WPE in UV LEDs.

## Supporting Information Available

Details on the epitaxial growth, process flow, and surface roughening, and the *I*–*V* characteristics before and after substrate removal (PDF)
