# Supplementary material for: Increased Light Extraction of Thin-Film Flip-Chip UVB LEDs by Surface Texturing
Source: ACS Photonics. 2023 Jan 24;10(2):368–73. doi: 10.1021/acsphotonics.2c01352 (PMC9936579; doi:10.1021/acsphotonics.2c01352)
Supplement: Supplementary file 1 — ph2c01352_si_002.pdf [file ph2c01352_si_002.pdf]

Supporting Information - Increased Light-Extraction of Thin-Film Flip-Chip UVB LEDs by Surface Texturing

Michael A. Bergmann[1], Johannes Enslin[2], Martin Guttmann[2], Luca Sulmoni[2], Neysha Lobo Ploch[3], Filip Hjort[1], Tim Kolbe[3], Tim Wernicke[2], Michael Kneissl[2,3], and Åsa Haglund[1]

[1]*Department of Microtechnology and Nanoscience, Chalmers University of Technology, 41296 Gothenburg, Sweden*
[2]*Institute of Solid State Physics, Technische Universität Berlin, 10623 Berlin, Germany*
[3]*Ferdinand-Braun-Institut gGmbH, Leibniz-Institut für Höchstfrequenztechnik, 12489 Berlin, Germany*

October 28, 2022

Pages S1 to S5
Figures S1 to S3

Supplementary Note 1: Epitaxial growth

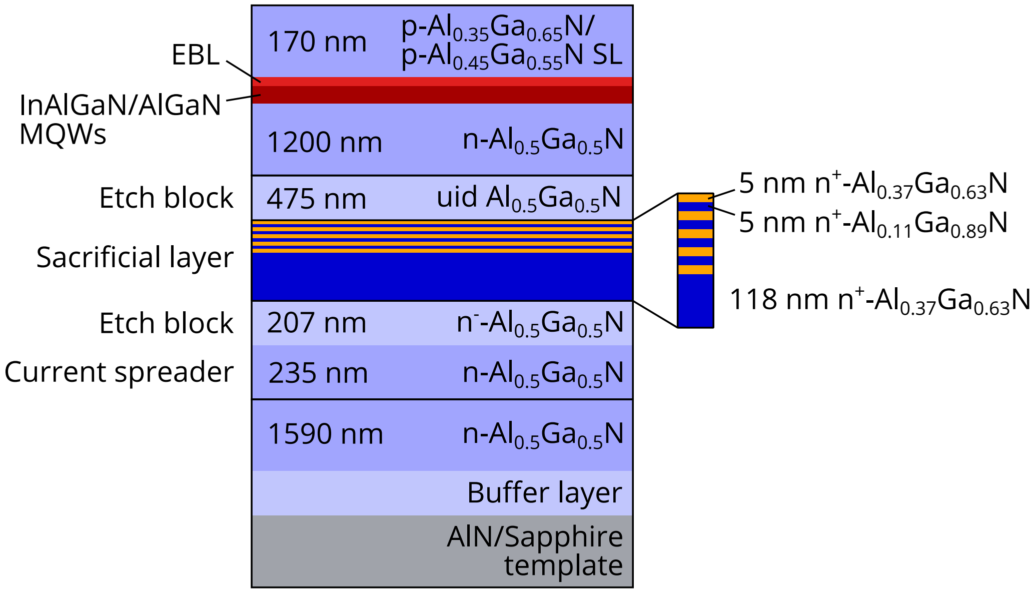

Figure S1: Epitaxial structure for fully-transparent TFFC-LEDs.

Figure S1 shows the epitaxial structure of the TFFC LEDs which was grown by metalorganic vapor-phase epitaxy in a close-coupled shower head reactor and the doping was achieved using silane (SiH_4) as an n-dopant and cyclopentadienylmagnesium (Cp_2Mg) as a p-dopant. In a first step, a pseudosubstrate was grown on a c-plane AlN/sapphire substrate with a relaxed $Al_{0.5}Ga_{0.5}N$ toplayer [1]. To later enable substrate removal, a 235 nm $Al_{0.5}Ga_{0.5}N$:Si current spreading layer for the electrochemical etching was grown. After that a multilayered sacrificial layer comprising a 118-nm thick $Al_{0.37}Ga_{0.63}N$ layer and a five-period structure of alternating 5-nm thick $Al_{0.11}Ga_{0.89}N$ and 5-nm thick $Al_{0.37}Ga_{0.63}N$ was grown in between two $Al_{0.50}Ga_{0.50}N$ etch block layers with a thickness of 207 nm and 475 nm respectively. Whereas the top etch block layer is unintentionally doped, the bottom etch block layer has an n-doping level of $0.5 \times 10^{18}\,cm^{-3}$ to enable current flow to the sacrificial layer. The Si concentration in the sacrificial layer was $2 \times 10^{19}\,cm^{-3}$ for all layers. On top of the sacrificial layer and etch block layer, the LED heterostructure was grown. This consisted of a 1200-nm-thick n-doped $Al_{0.5}Ga_{0.5}N$ current spreading layer (Si concentration: $2 \times 10^{18}\,cm^{-3}$)) followed by a threefold InAlGaN MQW active region, a 25-nm-thick $Al_{0.80}Ga_{0.20}N$ electron blocking layer, and a 170 nm Mg-doped $Al_{0.35}Ga_{0.65}N/Al_{0.45}Ga_{0.55}N$ superlattice. The threading dislocations density of the epitaxial layers is around $3 - 5 \times 10^9\,cm^{-2}$.

Supplementary Note 2: Process flow for TFFC LEDs

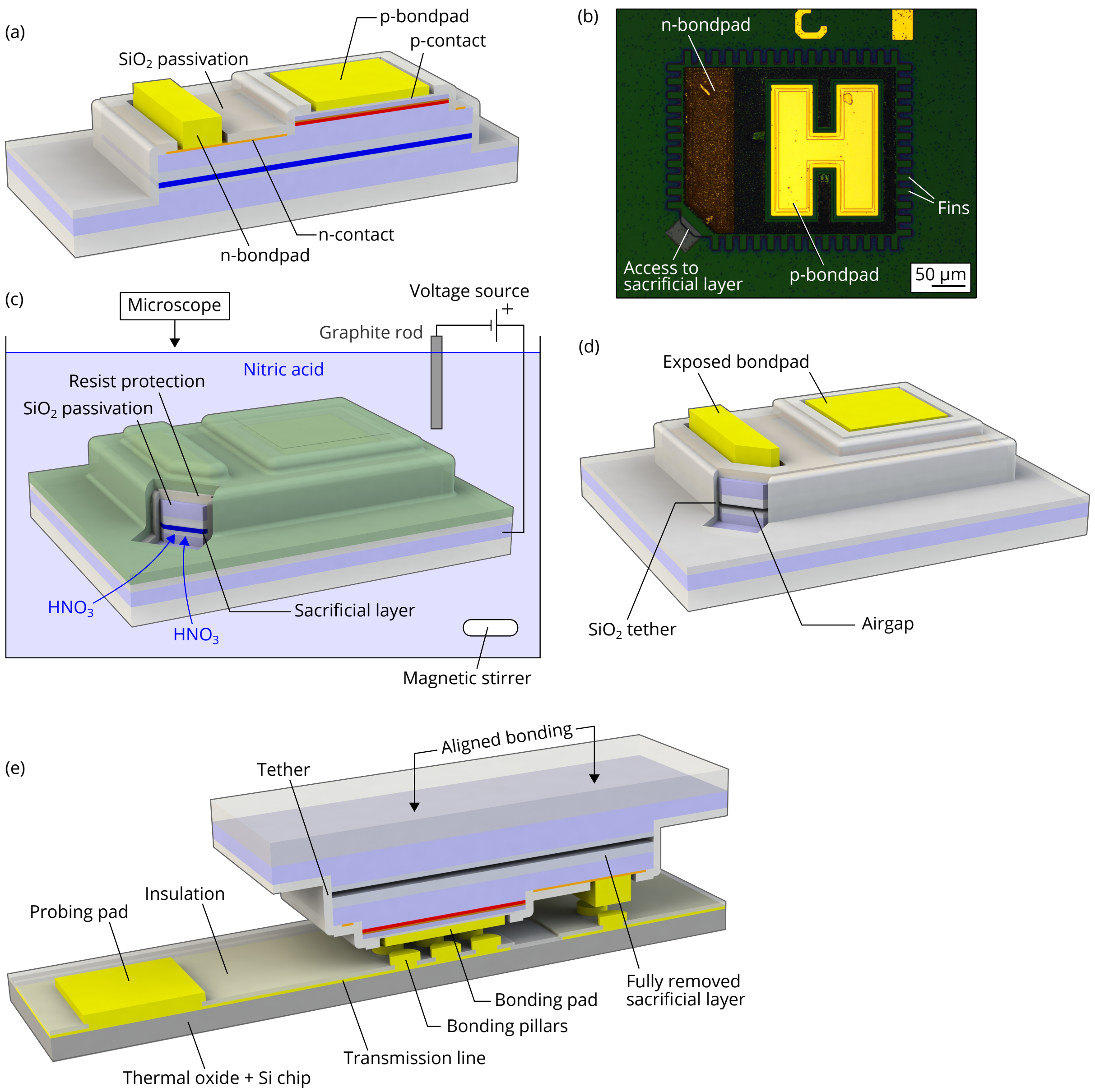

Figure S2: (a) Schematic and (b) microscope image of an LED after n- and p-side processing. (c) Schematic of the electrochemical etching setup and (d) schematic of an underetched LED. (e) Schematic of the aligned bonding process to transfer the LEDs.

The fabrication process started with the definition of the device mesa using Cl-based dry etching. The etching depth was chosen to fully expose the sacrificial layer. This was followed by a second dry etching step to define the lateral extent of the active region and to expose the n-side of the LED. Two different mesa shapes, a rectangular mesa and an interdigitated mesa, were fabricated to investigate the lateral current spreading and carrier injection. The V/Al/Ni/Au (15/90/20/30 nm) n-contact was evaporated and annealed for 40 s at 800°C in an N_2 atmosphere [2]. In the same process step, also the contact for applying the bias voltage during the electrochemical etching was formed. After that, a Pd/Al/Ni/Au (3/150/100/100 nm) p-contact was evaporated on the p-AlGaN. To improve the planarity of the devices for the bonding, the n- and p-contacts were leveled using a Ti/Au metal pad on the n-contact. Both contacts were finished by Ti/Au (10/600 nm) bondpads to provide a base for the thermocompression bonding. In the next step, a 500-nm thick SiO_2 layer was deposited by reactive sputtering and patterned using Ar/CHF_3-based dry etching (see Fig. S2(a)). This dielectric layer promoted unidirectional lateral

electrochemical etching, acted as a tether to keep the thin-film LEDs in place after the sacrificial layer removal and served as an insulation layer to prevent short-circuiting after the flip-chip bonding.

Before the sacrificial layer removal, a 3.5 μm-thick photoresist layer was deposited to protect the surface of the devices, especially the n-contact, from parasitic etching during the electrochemical etching. The thick resist layer further supports the SiO_2 tethers to keep devices in place. Figure S2(c) shows an LED at this stage, just before the electrochemical etching. The sacrificial layer was then laterally etched using a bias voltage of 15 V vs. a graphite rod in 0.3 M nitric acid. The etching was done at room temperature and the electrolyte was stirred using a magnetic stir bar without intentional illumination. The etching process was intentionally started from one corner of the device to proceed unidirectionally from this point beneath the device. The progress of the etching was monitored in-situ using an optical microscope to time the etching. After the complete removal of the sacrificial layer, the sample was immersed in de-ionized water to remove acid residues, followed by acetone and isopropanol cleaning. These solvents removed the resist protection and reduced the force on the membrane during the subsequent drying in air. After the cleaning process, the thin-film LEDs were held by the SiO_2 tether layer, see Fig. S2(d), which surrounds the device mesa. Because the tether area depends on the device perimeter, one group of devices included fins to increase the perimeter as shown in Fig. S2(b).

Figure S2(e) shows a schematic cross-section of the carrier chip onto which the thin-film LEDs were bonded. The carrier chip had electroplated Au-pillars of a height of 3 µm and diameter of 15 µm, which were arranged in a hexagonal pattern with a pitch of 30 µm. These pillars for the bonding were used instead of continuous large-area bonding pads to facilitate deformation and adaption during the thermocompression bonding [3]. Ti/Au metal lines were used to connect to the LEDs, which were electrically separated by the SiO_2-on-Si substrate and a SiO_2 layer on the top to further prevent potential short-circuiting of the LEDs after the flip-chip bonding.

Both the LED chips and the carrier chips were cleaned by UV-ozone to remove organic residues before the bonding. The thermocompression bond was accomplished under a mechanical pressure of around 100 MPa for 1 hour at 300°C in ambient air. After the bonding, during which the SiO_2 tethers break, the growth substrate of the UV LED is separated from the carrier chip while the single LEDs remain bonded to the carrier chip.

Individual LEDs were selectively roughened to investigate the impact of different degrees of roughness on the device performance. The lithography was selectively done using direct laser writing using a 5.3 μm-thick positive photoresist to avoid parasitic etching of other devices and device parts, especially the bonding and contact metal layer, during the roughening step. Because the TMAH-based developer (Microdeposit MF-CD-26) for the photoresist etches the N-polar AlGaN surface, a PMMA interlayer was spun before the photoresist to separate development and roughening. Therefore, after the resist development which stopped on the PMMA surface, O_2 plasma was used to remove the PMMA layer in the open area. These open areas were then exposed to the developer at room temperature for 9 minutes for one group of LEDs and 15 minutes for another group. After the roughening, the resist was stripped in solvents and O_2 plasma.

Supplementary Note 3: I-V characteristics

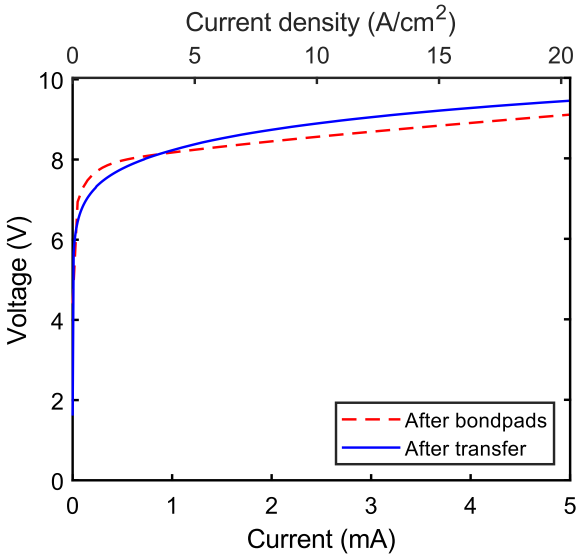

Figure S3: I-V-characteristics of an LED after bond pad fabrication and after sacrificial layer removal and transfer.

References

[1] Arne Knauer, Tim Kolbe, Jens Rass, Hyun Kyong Cho, Carsten Netzel, Sylvia Hagedorn, Neysha Lobo-Ploch, Jan Ruschel, Johannes Glaab, Sven Einfeldt, and Markus Weyers. High power UVB light emitting diodes with optimized n-AlGaN contact layers. *Jpn. J. Appl. Phys.*, 58(SC):SCCC02, 2019.

[2] Luca Sulmoni, Frank Mehnke, Anna Mogilatenko, Martin Guttmann, Tim Wernicke, and Michael Kneissl. Electrical properties and microstructure formation of V/Al-based n-contacts on high Al mole fraction n-AlGaN layers. *Photonics Res.*, 8(8):1381, 2020.

[3] C. Stölmacker, N. Lobo Ploch, A. Thies, S. Hochheim, J. Rass, F. Schnieder, A. Mogilatenko, J. Ruschel, T. Kolbe, S. Knigge, and S. Einfeldt. Electroplated Gold Microstuds for Thermocompression Bonding of UV LED Chips. *IEEE Trans. Components, Packag. Manuf. Technol.*, 9(12):2326–2331, 2019.
